# Lanthanide-Ion-Doping Effect on the Morphology and the Structure of NaYF_4_:Ln^3+^ Nanoparticles

**DOI:** 10.3390/nano12172972

**Published:** 2022-08-27

**Authors:** Nikita A. Bogachev, Anna A. Betina, Tatyana S. Bulatova, Viktor G. Nosov, Stefaniia S. Kolesnik, Ilya I. Tumkin, Mikhail N. Ryazantsev, Mikhail Yu. Skripkin, Andrey S. Mereshchenko

**Affiliations:** 1 Saint-Petersburg State University, 7/9 Universitetskaya emb., 199034 St. Petersburg, Russia; 2 Nanotechnology Research and Education Centre RAS, Saint Petersburg Academic University, 8/3 Khlopina Street, 194021 St. Petersburg, Russia

**Keywords:** lanthanides, rare earth, nanocrystals, hydrothermal synthesis, rare earth, nanoparticles, microparticles, crystal growth mechanism, NaYF_4_

## Abstract

Two series of β-NaYF_4_:Ln^3+^ nanoparticles (Ln = La–Nd, Sm–Lu) containing 20 at. % and 40 at. % of Ln^3+^ with well-defined morphology and size were synthesized via a facile citric-acid-assisted hydrothermal method using rare-earth chlorides as the precursors. The materials were composed from the particles that have a shape of uniform hexagonal prisms with an approximate size of 80–1100 nm. The mean diameter of NaYF_4_:Ln^3+^ crystals non-monotonically depended on the lanthanide atomic number and the minimum size was observed for Gd^3+^-doped materials. At the same time, the unit cell parameters decreased from La to Lu according to XRD data analysis. The diameter-to-length ratio increased from La to Lu in both studied series. The effect of the doping lanthanide(III) ion nature on particle size and shape was explained in terms of crystal growth dynamics. This study reports the correlation between the nanoparticle morphologies and the type and content of doping lanthanide ions. The obtained results shed light on the understanding of intrinsic factors’ effect on structural features of the nanocrystalline materials.

## 1. Introduction

Lanthanide-doped functional nanomaterials are widely studied due to their optical properties and broad application within such areas as the design of luminescent thermometers and photocatalysts, the development of sensors of biologically important substances and solar cells, single-molecule microscopy, solid-state lasers, and so on [1,2,3,4,5,6,7,8,9,10,11,12]. Such compounds allow making multifunctional materials through a combination of optical, magnetic, and other properties, which make them attractive and promising materials for theranostics. Combining the magnetic and luminescent properties of lanthanides provides a possibility to create bimodal materials for molecular imaging and non-invasive optical diagnostics of tissues of living organisms in vivo using magnetic resonance imaging (MRI), as well as materials for the detection and targeted treatment of cancer cells at early stages of the disease [13,14,15,16]. The optical and magnetic properties of doped rare-earth-element ions are well known to be sensitive in one’s own way to the host matrix type, crystal structure, morphology and size of substances, and rare-earth-element concentration [17,18,19,20,21,22,23,24,25,26,27,28,29].

The synthesis conditions provide the ability of targeted control of the aforementioned factors and, therefore, are regularly discussed by different research groups [30]. It is shown that usually rare-earth-ion doping is conducted simultaneously with the synthesis of host materials. The effectiveness of doping is determined by the size and valence of the host matrix and doped rare-earth ions and by the similarity of their reactivity. Fluoride compounds, such as NaREEF_4_ (REE = rare-earth element) and especially NaYF_4_, are one of the most popular materials to be used as a matrix for the fabrication of rare-earth-based nanomaterials. Depending on the synthesis conditions, NaYF_4_ crystallizes in two different polymorphic forms—cubic α-NaYF_4_ or hexagonal β-NaYF_4_ phase. It is known that the hexagonal phase is one of the most efficient host structures for the preparation of luminescent materials. There are different ways of preparing NaYF_4_-based nanoparticles [28]. Among them, hydrothermal synthesis seems to be more convenient because it makes the preparation safer, cost-efficient, environmentally friendly, and allows controlling the growth parameters of single-crystalline nanostructures. Generally, the morphology and the size of the final particles depend on the time of synthesis, temperature, pressure, pH of the precursor solution, ratio of the precursors, ion sources, and the composition of the solvent [31,32,33,34].

Chunxia Li et al. [35] showed the relationship between the architectural features of hexagonal NaYF_4_ nanocrystals and the conditions of preparation, specifically, different fluoride sources and pH values of the reaction system. It was shown that the fluoride sources and pH values of solutions of the initial reaction strongly effect the morphologies and dimensions of the final particles via changing their shape from prismatic microrods to hexagonal prismatic structures. To explain experimental results, authors have suggested that the ions in the reaction media are selectively adsorbed on the different crystal facets and this process directly depends on the pH of the medium. The difference in adsorption leads, ultimately, to the difference in crystal growth rate along different crystal axes. At the same time, the general structure motif is determined by the anisotropic structure of the thermodynamically stable β-phase NaYF_4_ seeds. Authors have additionally checked the influence of the synthesis time on the parameters of the final crystals. They found that the crystalline phase is dependent on the time of synthesis: hydrothermal treatment for 1 h leads to the formation of an unstable cubic α-NaYF_4_ phase, which slowly turns to a biphasic mixture of α-NaYF_4_ and hexagonal β-NaYF_4_ after 4 h of treatment, and, finally, up to 8 h, the cubic phase is totally replaced by a hexagonal form. Similar results can be found in other publications also. Bin Jiang and Xiangfu Wang [36] simulated the in situ growth process of β-NaYF_4_ crystal at a high temperature and pressure using the molecular dynamics theory. Their calculation predicts anisotropic growth leading to hexagonal form of crystals with a growth trend to rod shape that is in a good agreement with real experimental results. Other approaches to synthesis, for example, addition of surfactants or chelating agents into reaction media or using water-organic solvent leads to formation of crystals with controllable micro- and nanoscale sizes of different morphology: sphere, rod, prism, disk, octahedral [37,38]. This effect is caused by the influence of these additives on the nucleation rates because they change the diffusion of ions onto the surface of growing crystals. Another path to control the morphology of NaYF_4_ is a variation in precursors ratio. For example, it was found that the molar ratio of ions in reaction media can strongly affect the size and structure of crystals and leads to the formation of microcrystals with different shapes [39,40,41,42]. This phenomenon is probably connected with the effect of ions (primarily, F^−^ and chelating ions) on the relative growth rates of crystals along different axes. This explanation is related to the fact that during the hydrothermal synthesis, there are several simultaneous competitive processes. One of them is the formation (and following decomposition) of complexes of rare-earth ions with a chelating agent, and the second one is the asymmetric adhesion of ions on the facets of a growing crystal. The varying of the components’ ratio leads to a change in the balance between these processes and ultimately generates a range of different forms. The effect of lanthanide ions’ nature on crystal growth, morphology, and size of NaLnF_4_ (Ln = La − Yb) crystals is also known [43]. The varying of lanthanides leads to changing the particle morphology from rod to disk following the increasing of the ionic radius of Ln^3+^. It is connected with the anisotropic distribution of charge density on different planes of hexagonal structure and the difference in adsorption of organic additives on them. In one’s own way, this ion radius increase provides the reduction in surface energy required for NaLnF_4_ nucleation. Therefore, the size of crystals tends to become less from NaYbF_4_ to NaLaF_4_. In the case of lanthanide-doped nanomaterials, there arises a new aspect of the dopant nature’s influence on the structural characteristics of the final products. Due to the high affinity of rare-earth ions between each other and their ability to replace each other isomorphically in the crystal lattice, doped nanoparticles can be considered as solid solutions. Therefore, the parameters of the crystal lattice and the size of nanocrystals for a series of doped particles are expected to change monotonically on the whole range of compositions. At the same time, the shape of crystals is supposed to be constant in case the other conditions are equal. This prediction is confirmed by a large number of studies of materials based on several rare earth elements [39,44]. In our previous studies for a series of NaYF_4_:Eu^3+^, Gd^3+^ phosphors [45], we have shown also that the substitution of yttrium ions by europium ions, as well as in the case of simultaneous doping NaYF_4_ by Eu^3+^ and Gd^3+^, results in increase in the unit cell parameters of particles. The size reduction in particles is observed at the Eu^3+^ and Gd^3+^ concentration rise: ranging from 714 nm for NaYF_4_ to 94 nm for NaY_0.5_Eu_0.5_F_4_ and to 40 nm for NaGd_0.7_Eu_0.3_F_4_ samples. The nanoparticles appear in the shape of hexagonal prisms with a diameter/height ratio of about 1/1 and the shape does not change within the concentration range. We reported the similar results in other studies of Gd^3+^-doped NaYF_4_:Yb^3+^ and Er^3+^/Tm^3+^ microparticles [46]. In this case, also the addition of the Gd^3+^ dopant led to a decrease in the size of the obtained crystals. As an explanation, we assumed that the size of the crystals is guided by crystal growth rates. The larger positive-charge density in the Gd^3+^-containing crystal nucleus slows the diffusion of negatively charged fluoride ions, which leads to a reduction in the crystal growth rate and a smaller final size of Gd^3+^-co-doped microcrystals. This effect of Gd^3+^ doping is known and discussed in the literature, where the experimental observations are confirmed by first-principles calculations [47]. Moreover, it is noticed that gadolinium doping allows significantly decreasing the reaction temperature and time required for cubic-to-hexagonal phase conversion. In contrast with gadolinium effect, it is also known that the addition of a small amount of lutetium leads to a significant increase in the nanoparticle diameter with almost no change in height [48].

Despite the large and still growing number of papers devoted to the study of the effect of various factors on the growth mechanism, size, and morphology of nanoparticles, such works are mainly related to the research of the effect of the external conditions. At the same time, there is a lack of studies revealing the relationship between the nature of the doped lanthanide ions and the structural characteristics of the obtained nanoparticles under the condition where the external factors are equal. To fill this gap, in our work, we studied the impact of the nature of the lanthanide ions on the size and morphology of the NaYF_4_-doped nanoparticles.

## 2. Materials and Methods

Anhydrous chlorides of the rare-earth elements (YCl_3_, LaCl_3_, CeCl_3_, PrCl_3_, NdCl_3_, SmCl_3_, EuCl_3_, GdCl_3_, TbCl_3_, DyCl_3_, HoCl_3_, ErCl_3_, TmCl_3_, YbCl_3_, LuCl_3_, 99.999%) were purchased from Chemcraft (Kaliningrad, Russia), NaOH, NH_4_F, citric acid, and ethanol were purchased from Sigma-Aldrich Pty Ltd. (Darmstadt, Germany), and used without additional purification.

Microcrystalline β-NaYF_4_ samples co-doped with Ln^3+^ were synthesized via the hydrothermal method using citric acid as a stabilizing agent. The common synthesis routine is described below. Rare-earth chlorides taken in stoichiometric amounts (total amount of rare-earth chlorides was 0.75 mmol) with 3 mmol of citric acid were dissolved in distilled water to obtain 5 mL solution in total. Then, 2.5 mL of an aqueous solution containing 9 mmol of NaOH was added to the flask of the previous solution. After vigorous stirring for 30 min, 8 mL of aqueous solution containing 11 mmol of NaOH and 11 mmol of NH_4_F was added into the above solution. The solution was maintained after vigorous stirring for 30 min at room temperature before being transferred to a Teflon-lined autoclave with an internal volume of 20 mL and heated for 17 h at the temperature of 180 °C. After that, the precipitate was separated from the reaction mixture by centrifugation, washed with ethanol and deionized water, and dried at 60 °C for 24 h. The desired microstructure materials were obtained in the form of white powders. In this work, we synthesized and studied two series of substances: NaY_0.8_Ln_0.2_F_4_ and NaY_0.6_Ln_0.4_F_4_ (Ln = La–Nd, Sm–Lu).

The morphologies of microstructures of the synthesized samples were characterized using scanning electron microscopy (SEM) on a Zeiss Merlin electron microscope (Zeiss, Jena, Germany) using an energy-dispersive X-ray spectroscopy (EDX) module (Oxford Instruments INCAx-act, Abingdon, UK). X-ray powder diffraction (XRD) measurements were performed on a D2 Phaser (Bruker, Billerica, MA, USA) X-ray diffractometer using Cu Kα radiation (λ = 1.54056 Å). The content of rare-earth ions was confirmed by EDX spectroscopy.

## 3. Results and Discussion

### 3.1. Crystal Structure

**Figure 1:**
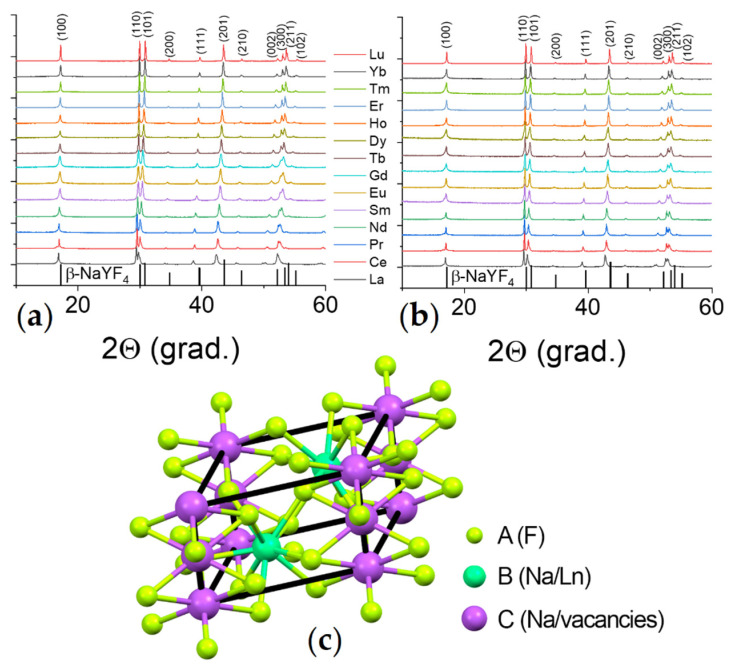
XRD patterns of (**a**) NaY_0.8_Ln_0.2_F_4_ and (**b**) NaY_0.6_Ln_0.4_F_4_ (Ln = La–Lu); (**c**) the fragment of NaYF_4_ crystal structure.

The X-ray powder diffraction (XRD) patterns are shown in Figure 1. Analysis of XRD patterns demonstrates that all synthesized materials of both series (NaY_0.8_Ln_0.2_F_4_ and NaY_0.6_Ln_0.4_F_4_) have the same crystalline phase, which corresponds to the hexagonal β-NaYF_4_ (JCPDS № 16-0334). Additional diffraction peaks corresponding to the impurities were not observed. Thus, our data demonstrate that Y^3+^ ions are isomorphically substituted by Ln^3+^ ions in NaYF_4_ materials. The unit cell structure of β-NaYF_4_ is shown in Figure 1 [49]. In this structure, one can observe three positions of atoms: A, B, and C. Sodium atoms and vacancies are located at the position C (coordination number is 9) in a ratio 1:1. Sodium and yttrium(III) ions occupy position B (coordination number is 6) in a ratio 1:3. The stoichiometry of the unit cell is Na_1.5_Y_1.5_F_6_.

**Figure 2:**
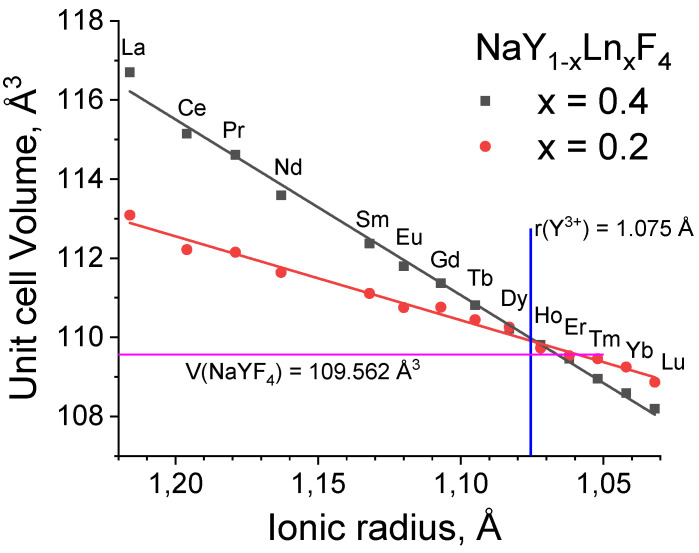
The dependence of unit cell volumes of NaY_0.8_Ln_0.2_F_4_ and NaY_0.6_Ln_0.4_F_4_ (Ln = La–Nd, Sm–Lu) on the ionic radii of Ln^3+^ ions (the coordination number is equal to 9). The ionic radius of Y^3+^ is shown as blue line for comparison. The unit cell volume of NaYF_4_ is shown as magenta line.

Unit cell parameters were refined from the XRD patterns by the Rietveld method. The dependence of the unit cell volume on the Ln^3+^ ion radius is shown in Figure 2 and in Tables S1 and S2, Supplementary Materials. Unit cell volumes linearly depend on the ionic radius of the doping lanthanide(III) ion, which decreases from La to Lu [50]. We found that unit cell volumes of NaY_0.8_Ln_0.2_F_4_ samples are in the range from 108 to 113 Å^3^, while for NaY_0.6_Ln_0.4_F_4,_ this range is a bit wider (108–117 Å^3^), because NaY_0.6_Ln_0.4_F_4_ materials contain more ions with larger ionic radii than that of Y^3+^.

### 3.2. Morphology

**Figure 3:**
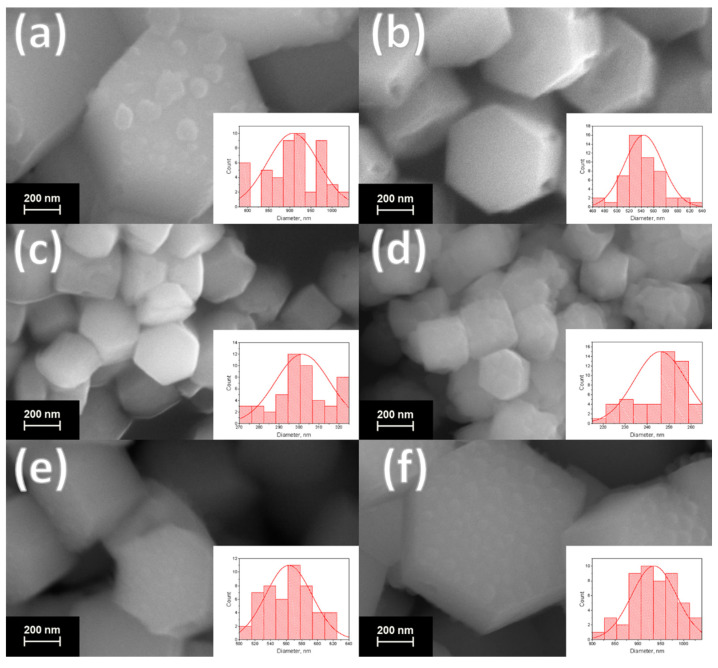
SEM images of the samples: (**a**) NaY_0.8_La_0.2_F_4_, (**b**) NaY_0.8_Pr_0.2_F_4_, (**c**) NaY_0.8_Sm_0.2_F_4_, (**d**) NaY_0.8_Gd_0.2_F_4_, (**e**) NaY_0.8_Ho_0.2_F_4_, (**f**) NaY_0.8_Lu_0.2_F_4_. Particle size distribution is shown in the insets. The average diameter of the particles is equal to 907 ± 61, 544 ± 31, 302 ± 14, 246 ± 12, 563 ± 30 and 935 ± 49 nm for the La^3+^, Pr^3+^, Sm^3+^, Gd^3+^, Ho^3+^ and Lu^3+^, respectively.

**Figure 4:**
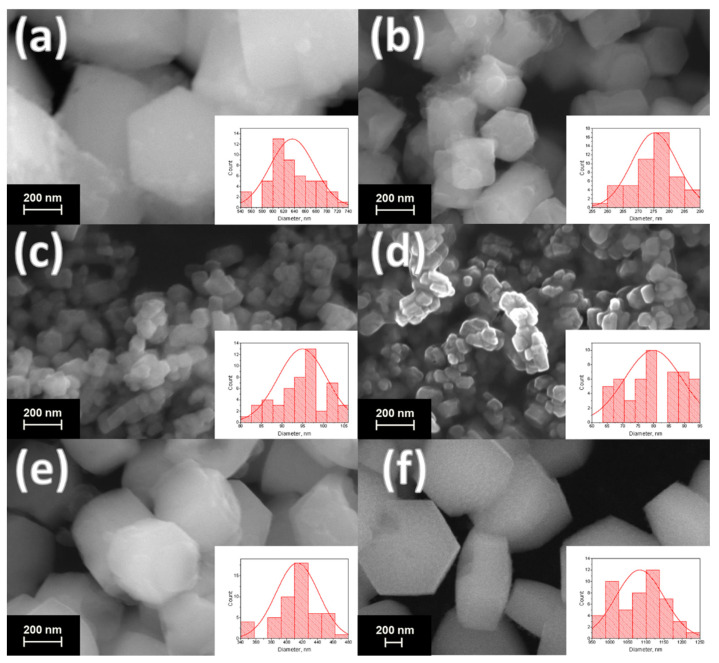
SEM images of the samples: (**a**) NaY_0.6_La_0.4_F_4_, (**b**) NaY_0.6_Pr_0.4_F_4_, (**c**) NaY_0.6_Sm_0.4_F_4_, (**d**) NaY_0.6_Gd_0.4_F_4_, (**e**) NaY_0.6_Ho_0.4_F_4_, (**f**) NaY_0.6_Lu_0.4_F_4_. Particle size distribution is shown in the insets. The average diameter of the particles is equal to 636 ± 41, 95 ± 6, 81 ± 4, 80 ± 9, 177 ± 8 and 1083 ± 67 nm for the La^3+^, Pr^3+^, Sm^3+^, Gd^3+^, Ho^3+^ and Lu^3+^, respectively.

**Figure 5:**
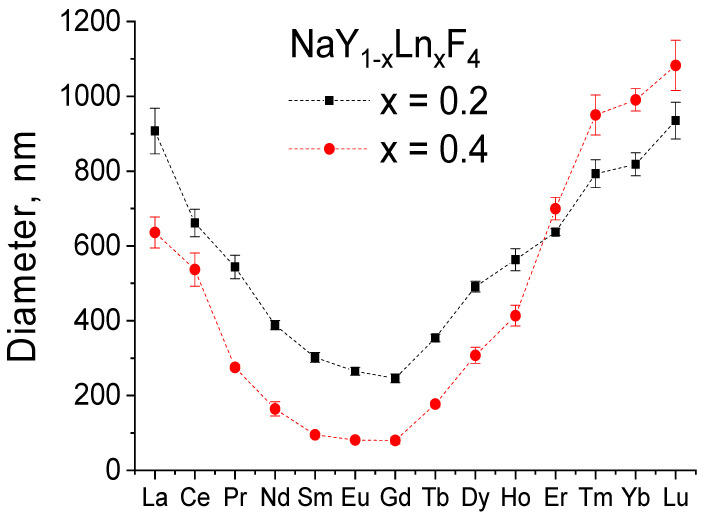
The average particle diameter of NaY_0.8_Ln_0.2_F_4_ and NaY_0.6_Ln_0.4_F_4_ (Ln = La–Lu) materials.

**Figure 6:**
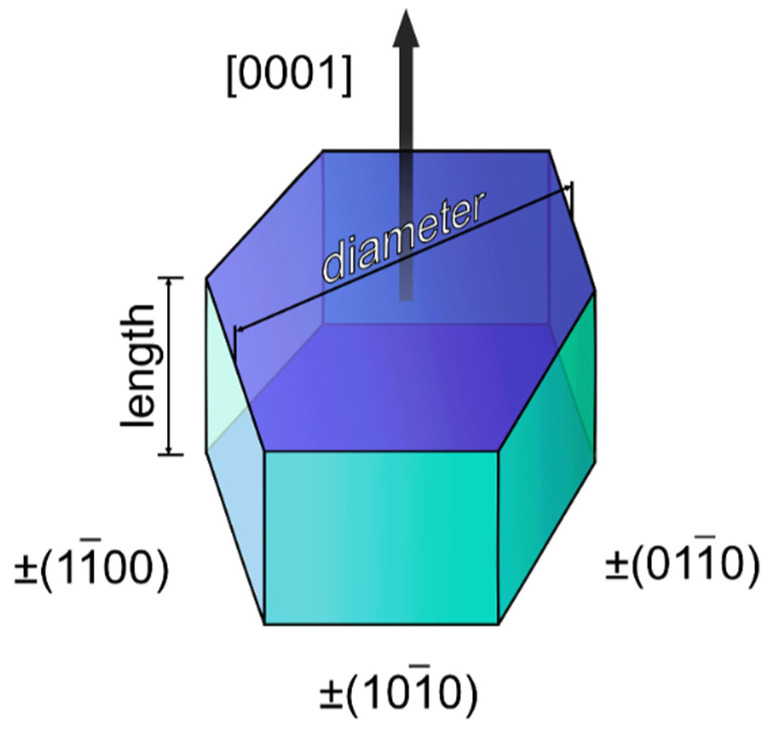
The shape and the anisotropy of the hexagonal prisms of β-NaYF_4_.

The scanning electron microscopy (SEM) images of the synthesized materials are given in Figure 3 and Figure 4 (main text) and in Figures S1 and S2 (Supplementary Materials). The particle diameters were obtained from SEM images, and the size distribution is shown in the insets of Figure 3 and Figure 4 (main text) and in Figures S3 and S4 (Supplementary Materials). The mean diameter of the particle was calculated from this distribution and is given in the legends of Figure 3 and Figure 4 and in Tables S3 and S4 in Supplementary Materials. The particles have a shape of hexagonal prisms and size between 80 and 1100 nm depending on composition. The particle size nonmonotonically changes in series NaY_0.8_Ln_0.2_F_4_ and NaY_0.6_Ln_0.4_F_4_ regarding the atomic number of the doping lanthanide, Figure 5. The average particle diameter decreases from La to Gd, where it reaches minimum value, and then further increases from Gd to Lu. These observations can be explained from the dynamics of the crystal growth. The crystal size is determined by the ratio of nucleation and crystal growth rates. If the nucleation rate is larger than crystal growth rate, the small single crystals are formed. In the opposite case, when nucleation is slow, but crystal growth is fast, the large single crystals are formed. Na^+^ and citrate (Cit^3−^) ions, which are in great excess in the reaction mixture (Ln^3+^:Cit^3−^:Na^+^ = 1:4:27), can be adsorbed on different faces of the crystals/crystal grains. Na^+^ ions are predominantly adsorbed on [0001] the prismatic top/bottom crystal plane, and Cit^3−^ ions are adsorbed more on $\left[10\bar{1}0\right]$ side facets than on [0001] ones [51,52], Figure 6. The adsorbed Na^+^ and Cit^3−^ ions inhibit crystal growth along corresponding directions. Hence, the decrease in unit cell volume results in the slowing down the crystal growth until the surface is fully covered by these ions. Therefore, the crystal growth rate effect is probably stronger pronounced in the beginning of the period. As was abovementioned, the unit cell volumes decrease in obtained samples from La to Lu. The smaller unit cell volumes correspond to the higher surface charge densities, which results in stronger adsorption of growth-inhibiting Na^+^ and Cit^3−^ ions on the NaY_1−*x*_Ln*_x_*F_4_ crystal grains resulting in slowing down the crystal growth rate. At the same time, Sun et al., reported [43] that NaLnF_4_ nucleation rate is higher for the lanthanides of larger ionic radius, and, therefore, should decrease from La to Lu. Thus, the observed particle size decrease from La to Gd is dominated by the decrease in crystal growth rate due to the adsorption of Na^+^ and Cit^3−^ ions inhibiting crystal growth. At some point, probably for Gd-doped species, the large amount of Na^+^ and Cit^3−^ ions covers the crystal grain surface, and additional Na^+^ and Cit^3−^ adsorption is not favorable anymore. Therefore, from Gd to Lu, the crystal growth rate changes insignificantly. At the same time, the nucleation rate monotonically drops from La to Lu, which results in a rise in the crystal growth to nucleation rates ratio that leads to particle size increase from Gd to Lu. Comparing the particle sizes between NaY_0.8_Ln_0.2_F_4_ and NaY_0.6_Ln_0.4_F_4_ series, one can notice that between La and Ho, the materials containing 20 at. % of dopant are larger than samples containing 40 at. % of Ln^3+^. NaY_0.8_Er_0.2_F_4_ and NaY_0.6_Er_0.4_F_4_ particles have the comparable diameters of 637 ± 9 and 700 ± 30 nm, respectively, which are also close to the diameters of NaYF_4_ particles synthesized by the same method [45], 714 ± 35 nm. Er^3+^ and Y^3+^ ions have similar ionic radii of 1.062 and 1.075 Å, which results in closed unit cell volumes of NaY_0.8_Er_0.2_F_4_ (109.538 Å^3^), NaY_0.6_Er_0.4_F_4_ (109.456 Å^3^) (Figure 2), and NaYF_4_ (109.562 Å^3^) and in the same electrostatic behavior as the abovementioned materials. For Tm, Yb, and Lu, the opposite situation is observed: the materials containing 40 at. % of dopant are larger than samples containing 20 at. % of Ln^3+^. These observations are consistent with unit cell volumes values (Figure 2), where crossing of the curves corresponding to NaY_0.8_Ln_0.2_F_4_ and NaY_0.6_Ln_0.4_F_4_ is observed at Ho or Er; therefore, one can suppose that inversion between sizes of NaY_0.8_Ln_0.2_F_4_ and NaY_0.6_Ln_0.4_F_4_ also can be explained in terms of atomic radii of Ln^3+^. Indeed, as we mentioned previously, lanthanide ions with large atomic radii demonstrate a tendency towards faster nucleation. For the NaY_1-*x*_Ln*_x_*F_4_ (Ln = La–Dy) series, nucleation is limited by Ln^3+^ ions, which form grains faster than Y^3+^ ions. Furthermore, this grain growth is determined by the presence of Y^3+^ ions. Hence, a higher concentration of Ln^3+^ ions (Ln = La–Dy) results in the formation of smaller particles. This explanation is consistent with data reported in previous works. Thus, previously, we reported that for NaEu*_x_*Y_1-*x*_F_4_ materials [45], increasing the Eu^3+^ concentration from 0 to 50 at. % results in a particle size decrease from (714 ± 35) nm to (94 ± 5) nm. When the ionic radii of lanthanides are comparable, all ions form grains with similar rates. Indeed, in the NaEu*_x_*Gd_1−*x*_F_4_ series, where Gd^3+^ and Eu^3+^ have close values of ionic radii (r(Gd^3+^) = 1.107 Å, r(Eu^3+^) = 1.12 Å), nanoparticles have similar sizes [53]. Hence, the particle size increases simultaneously with the increase the Ln^3+^ content when the ionic radii of doping lanthanides are lower than the ionic radius of yttrium (III) ion in series NaY_1-*x*_Ln*_x_*F_4_ (Ln = Tm–Lu).

**Figure 7:**
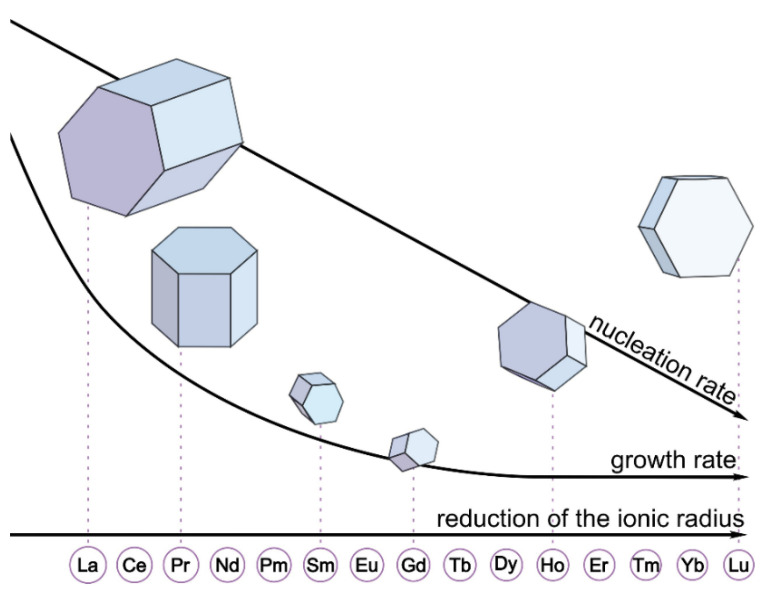
The effect of the crystal growth factors on the particle morphology of NaYF_4_:Ln^3+^.

Additionally, one can notice that particle shape depends on the dopant atomic number: the diameter-to-length ratio increases from La to Lu in both NaY_0.8_Ln_0.2_F_4_ and NaY_0.6_Ln_0.4_F_4_ series (Figure 3 and Figure 4 in main text and in Figures S1 and S2 in Supplementary Materials). Thus, NaYF_4_:La^3+^ particles have a shape of elongated prisms or “rods” (Figure 3a and Figure 4a), but NaYF_4_:Lu^3+^ particles are observed as flattened prisms or “tablets” (Figure 3f and Figure 4f). As was abovementioned, Na^+^ and Cit^3−^ ions are predominantly adsorbed on prismatic top/bottom [0001] and $\left[10\bar{1}0\right]$ side crystal planes, correspondingly, and inhibit the crystal growth in these directions. The surface charge densities increase from La to Lu due to the unit cell volume reduction, which results in stronger adsorption of growth-inhibiting Na^+^ and Cit^3−^ ions on the NaMF_4_ crystal grains (M = Y/Ln). We believe that charge densities increase effects on sodium ion adsorption to a greater degree than citrate ions, because Na^+^ is a small ion that has high positive charge density, but the negative charge of a citrate ion is delocalized along the citrate ion π-system. Therefore, the surface charge densities’ increase from La to Lu to a greater degree slows down the crystal growth along longitudinal direction [0001] than along the transverse one $\left[10\bar{1}0\right]$, which results in a flattening of the hexagonal-prism-shaped particles in the end of the period. For a better understating of above-described connection between growth factors and transformation of particles’ shape and size, a schematic illustration of the proposed explanation is given in Figure 7.

## 4. Conclusions

In this work, we reported the morphology and crystal size dependence on the nature of the doping lanthanide(III) ions in NaY_0.8_Ln_0.2_F_4_ and NaY_0.6_Ln_0.4_F_4_ series. The reported materials were synthesized by the one-pot hydrothermal method in an autoclave by heating at the temperature of 180 °C within 17 h. Analysis of XRD patterns demonstrated that all synthesized samples have a β-NaYF_4_ crystal structure. The unit cell parameters were calculated from XRD by the Rietveld method. It was demonstrated that the unit cell volume decreases from La to Lu simultaneously with Ln^3+^ ionic radii. According to scanning electron microscopy data, the particles of all the studied materials have a shape of hexagonal prisms and size of 80–1100 nm depending on the sample composition. The particle size nonmonotonically changes in both series as the atomic number of the doping lanthanide increases. The average particle diameter decreases from La to Gd, where it reaches the minimum value, and then further increases from Gd to Lu. Additionally, the particle shape depends on the dopant atomic number: the diameter-to-length ratio increases from La to Lu in both NaY_0.8_Ln_0.2_F_4_ and NaY_0.6_Ln_0.4_F_4_ series. The effect of the nature of the doping lanthanide(III) ion on particle size and shape was explained in terms of crystal growth dynamics, the relationship between the atomic number of the Ln^3+^ ion and the surface charge density, and the role of sodium and citrate ions presented in the reaction mixture in excess. 

## Data Availability

The data presented in this study are available in Supplementary Materials.

## Supplementary Materials

The following supporting information can be downloaded at: https://www.mdpi.com/article/10.3390/nano12172972/s1, Figure S1: SEM images of the NaY_0.8_Ln_0.2_F_4_ samples, Ln = (a) La (b) Ce (c) Pr (d) Nd (e) Sm (f) Eu (g) Gd (h) Tb (i) Dy (j) Ho (k) Er (l) Tm (m) Yb (n) Lu; Figure S2: SEM images of the NaY_0.6_Ln_0.4_F_4_ samples, Ln = (a) La (b) Ce (c) Pr (d) Nd (e) Sm (f) Eu (g) Gd (h) Tb (i) Dy (j) Ho (k) Er (l) Tm (m) Yb (n) Lu; Figure S3: Particle diameter distribution of the NaY_0.8_Ln_0.2_F_4_ samples, Ln = (a) La (b) Ce (c) Pr (d) Nd (e) Sm (f) Eu (g) Gd (h) Tb (i) Dy (j) Ho (k) Er (l) Tm (m) Yb (n) Lu; Figure S4: Particle diameter distribution of the NaY_0.6_Ln_0.4_F_4_ samples, Ln = (a) La (b) Ce (c) Pr (d) Nd (e) Sm (f) Eu (g) Gd (h) Tb (i) Dy (j) Ho (k) Er (l) Tm (m) Yb (n) Lu; Table S1: Unit cell parameters of the NaY_0.8_Ln_0.2_F_4_ samples; Table S2: Unit cell parameters of the NaY_0.6_Ln_0.4_F_4_ samples; Table S3: Mean particle diameter of the NaY_0.8_Ln_0.2_F_4_ samples; Table S4: Mean particle diameter of the NaY_0.6_Ln_0.4_F_4_ samples.
