# Supplementary material for: Lanthanide-Ion-Doping Effect on the Morphology and the Structure of NaYF4:Ln3+ Nanoparticles
Source: Nanomaterials (Basel). 2022 Aug 27;12(17):2972. doi: 10.3390/nano12172972 (PMC9457563; doi:10.3390/nano12172972)
Supplement: Supplementary file 1 [file nanomaterials-12-02972-s001.zip › nanomaterials-1878106-supplementary.pdf]

Supplementary Materials

Lanthanide-Ion-Doping Effect on the Morphology and the Structure of $NaYF_4:Ln^{3+}$ Nanoparticles

Nikita A. Bogachev [1], Anna A. Betina [1], Tatyana S. Bulatova [1], Viktor G. Nosov [1], Stefaniia S. Kolesnik [1], Ilya I. Tumkin [1], Mikhail N. Ryazantsev [1,2], Mikhail Yu. Skripkin [1] and Andrey S. Mereshchenko [1,*]

[1] Saint-Petersburg State University, 7/9 Universitetskaya emb., 199034 St. Petersburg, Russia
[2] Nanotechnology Research and Education Centre RAS, Saint Petersburg Academic University, 8/3 Khlopina Street, 194021 St. Petersburg, Russia
* Correspondence: a.mereshchenko@spbu.ru; Tel.: +7-951-677-5465

The supplementary materials contain unit cell parameters, refined from XRD patterns, SEM images of the synthesized samples, particle size distribution and mean particle size with standard deviation of samples.

Table S1. Unit cell parameters of the $NaY_{0.8}Ln_{0.2}F_4$ samples.

Ln	a, Å	c, Å	V, Å
La	6.0316	3.5895	113.09
Ce	6.0224	3.5727	112.219
Pr	6.0223	3.5706	112.15
Nd	6.0172	3.5604	111.64
Sm	6.0108	3.55105	111.11
Eu	6.0057	3.5456	110.751
Gd	6.0073	3.544	110.76
Tb	6.0031	3.53876	110.443
Dy	6.0014	3.5348	110.258
Ho	5.9926	3.52809	109.726
Er	5.99068	3.52434	109.538
Tm	5.99048	3.52209	109.461
Yb	5.98698	3.51937	109.248
Lu	5.98162	3.51328	108.864

Table S2. Unit cell parameters of the $NaY_{0.6}Ln_{0.4}F_4$ samples.

Ln	a, Å	c, Å	V, Å
La	6.0736	3.6531	116.703
Ce	6.0569	3.6242	115.145
Pr	6.0544	3.6105	114.612
Nd	6.0417	3.59336	113.591
Sm	6.0254	3.5739	112.369
Eu	6.0171	3.5659	111.802
Gd	6.0125	3.5573	111.368
Tb	6.0064	3.54682	110.815
Dy	5.99899	3.53639	110.218

Ho	5.99391	3.52931	109.811
Er	5.98968	3.5229	109.456
Tm	5.98275	3.51481	108.953
Yb	5.97786	3.50891	108.592
Lu	5.97286	3.50182	108.191

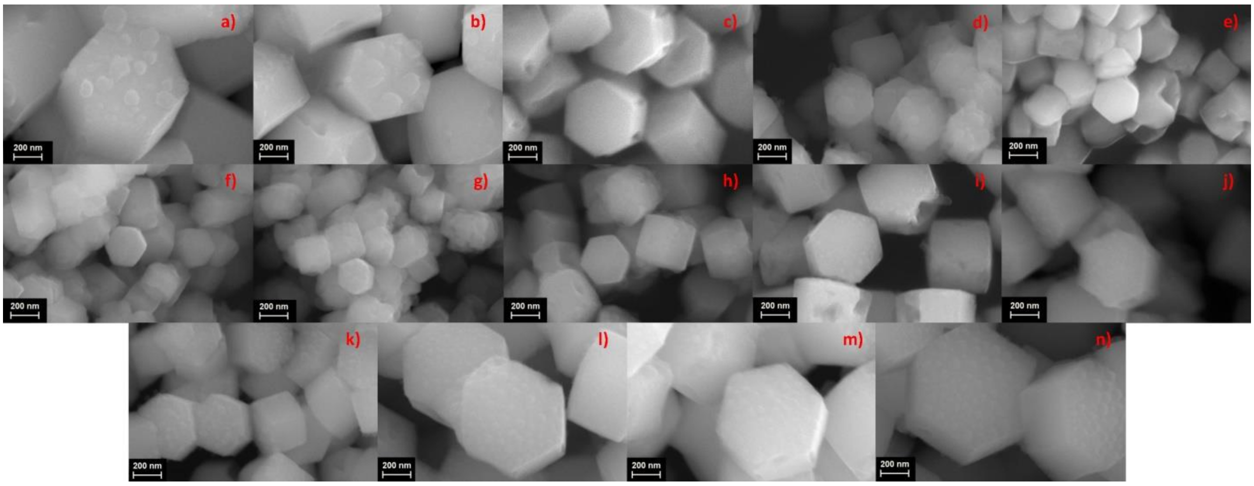

Figure S1. SEM images of the $NaY_{0.8}Ln_{0.2}F_4$ samples, Ln = (**a**) La (**b**) Ce (**c**) Pr (**d**) Nd (**e**) Sm (**f**) Eu (**g**) Gd (**h**) Tb (**i**) Dy (**j**) Ho (**k**) Er (**l**) Tm (**m**) Yb (**n**) Lu.

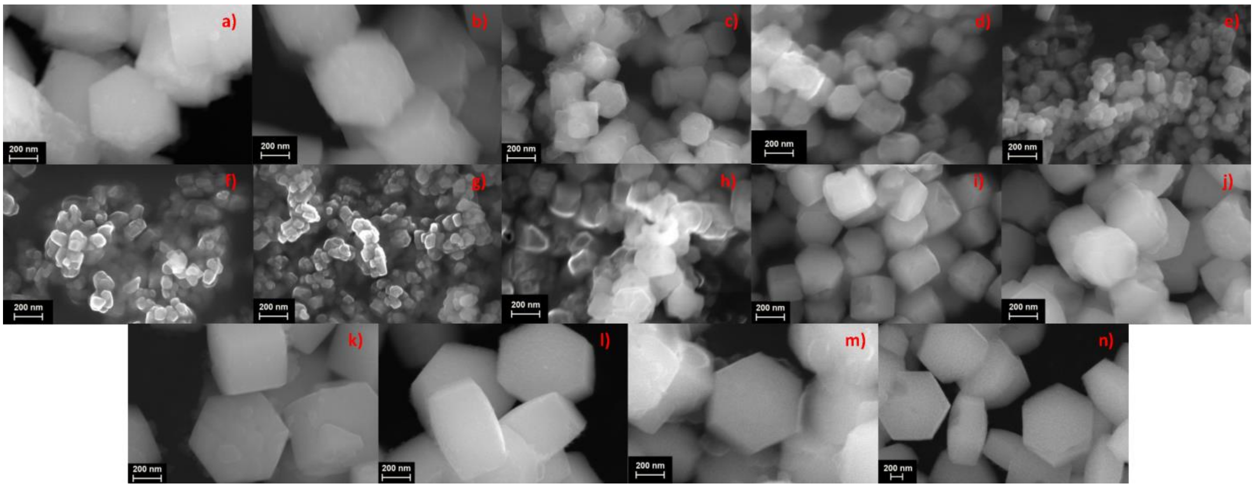

Figure S2. SEM images of the $NaY_{0.6}Ln_{0.4}F_4$ samples, Ln = (**a**) La (**b**) Ce (**c**) Pr (**d**) Nd (**e**) Sm (**f**) Eu (**g**) Gd (**h**) Tb (**i**) Dy (**j**) Ho (**k**) Er (**l**) Tm (**m**) Yb (**n**) Lu.

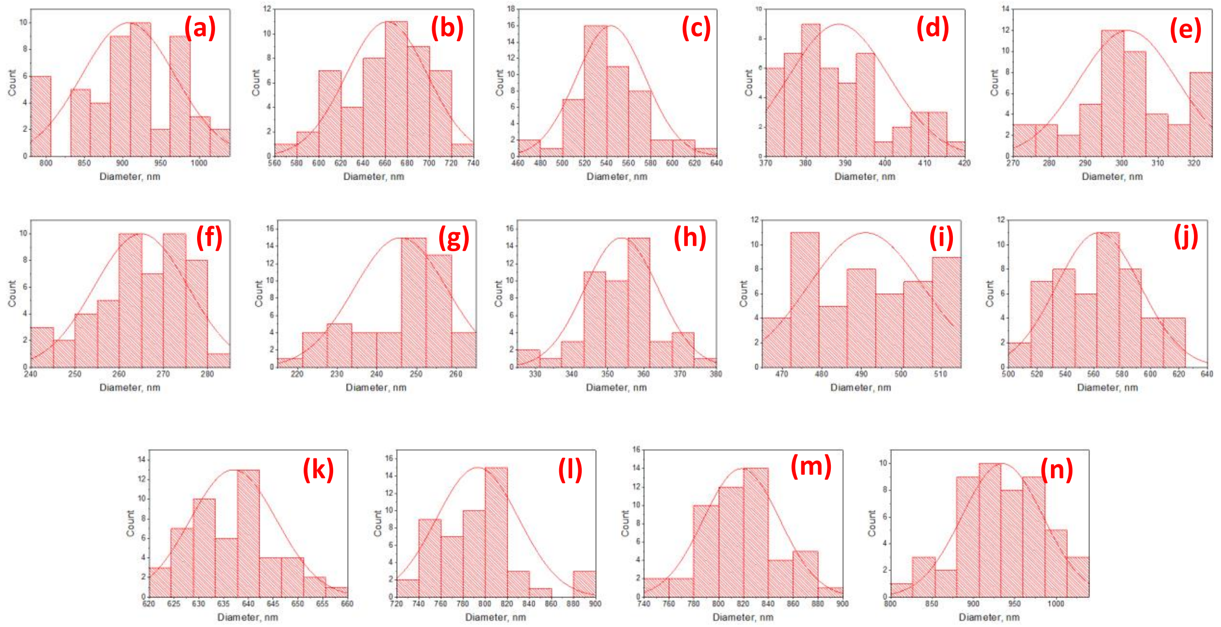

Figure S3. Particle diameter distribution of the $NaY_{0.8}Ln_{0.2}F_4$ samples, Ln = (**a**) La (**b**) Ce (**c**) Pr (**d**) Nd (**e**) Sm (**f**) Eu (**g**) Gd (**h**) Tb (**i**) Dy (**j**) Ho (**k**) Er (**l**) Tm (**m**) Yb (**n**) Lu.

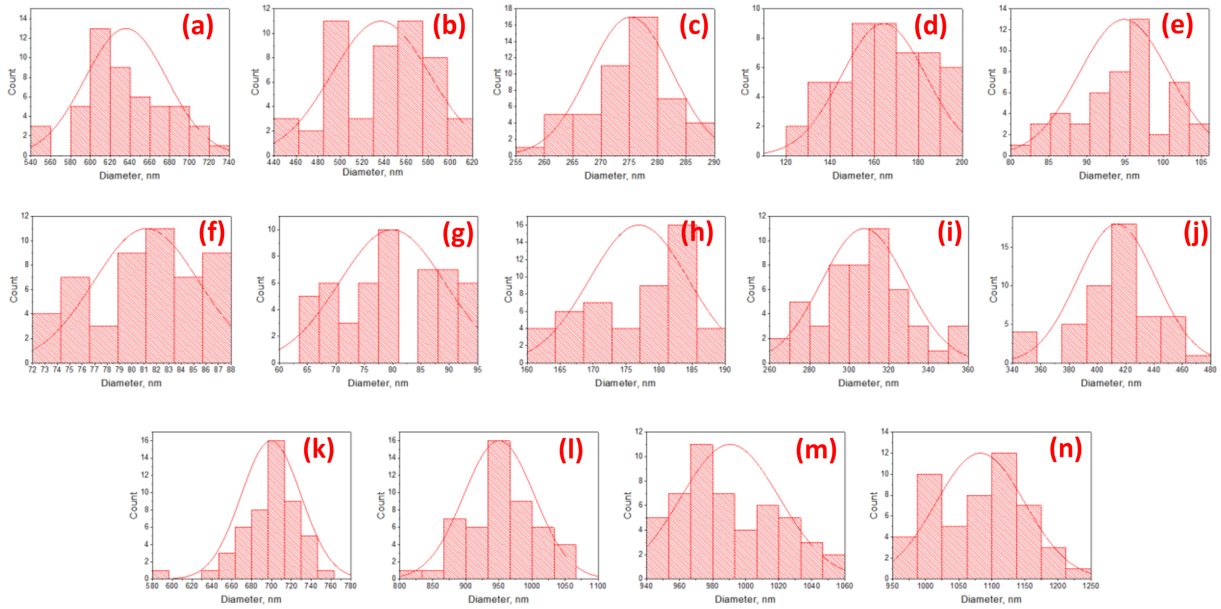

Figure S4. Particle diameter distribution of the $NaY_{0.6}Ln_{0.4}F_4$ samples, Ln = (**a**) La (**b**) Ce (**c**) Pr (**d**) Nd (**e**) Sm (**f**) Eu (**g**) Gd (**h**) Tb (**i**) Dy (**j**) Ho (**k**) Er (**l**) Tm (**m**) Yb (**n**) Lu.

Table S3. Mean particle diameter of the $NaY_{0.8}Ln_{0.2}F_4$ samples.

Ln	Diameter, nm	Standard deviation, nm
La	907	61
Ce	661	37
Pr	544	31
Nd	388	13
Sm	302	14
Eu	265	11
Gd	246	12
Tb	354	10
Dy	491	14
Ho	563	30
Er	637	9
Tm	793	37
Yb	818	31
Lu	935	49

Table S4. Mean particle diameter of the $NaY_{0.6}Ln_{0.4}F_4$ samples.

Ln	Diameter, nm	Standard deviation, nm
La	636	41
Ce	537	44
Pr	275	7
Nd	164	19
Sm	95	6
Eu	81	4
Gd	80	9
Tb	177	8
Dy	307	21
Ho	414	28
Er	700	30
Tm	950	54
Yb	991	30
Lu	1083	67
